# FOPR test: a virtual reality-based technique to assess field of perception and field of regard in hemispatial neglect

**DOI:** 10.1186/s12984-021-00835-1

**Published:** 2021-02-18

**Authors:** Tae-Lim Kim, Kwanguk Kim, Changyeol Choi, Ji-Yeong Lee, Joon-Ho Shin

**Affiliations:** 1 Department of Rehabilitation Medicine, National Rehabilitation Center, Ministry of Health and Welfare, Seoul, Republic of Korea; 2 Department of Computer Science, Hanyang University, Seoul, Republic of Korea; 3 Department of Neurorehabilitation, National Rehabilitation Center, 58, Samgaksan-ro, Gangbuk-gu, Seoul, 01022 Republic of Korea

**Keywords:** Field of perception, Field of regard, Hemispheric stroke, Virtual reality, Rehabilitation, Visuospatial function

## Abstract

**Background:**

We previously proposed a novel virtual reality-based method to assess human field of perception (FOP) and field of regard (FOR), termed the FOPR test. This study assessed the diagnostic validity of the FOPR test for hemispatial neglect (HSN).

**Methods:**

We included 19 stroke patients with a lesion in the right hemisphere and with HSN (HSN+SS), 22 stroke patients with a lesion in the right hemisphere and without HSN (HSN−SS), and 22 healthy controls aged 19–65 years. The success rate (SR) and response time (RT) in the FOPR test for both FOP and FOR were assessed (FOP-SR, FOR-SR, FOP-RT, and FOR-RT, respectively). Using a Bland–Altman plot, agreements between the FOPR test and conventional tests were confirmed, and the FOPR test accuracy was verified using the support vector machine (SVM). Measured values were analysed using ANOVA and Kruskall–Wallis tests for group comparison.

**Results:**

The Bland–Altman plot showed good agreement between FOPR test and conventional tests; individuals within 95% agreement limits were within the range of 94.8–100.0%. The SVM classification accuracy, using FOP and FOR variables from the left hemispace, ranged from 83.3 to 100.0% in a binary classification (HSN vs non-HSN). The FOPR test demonstrated differences in SR and RT for both FOP and FOR across the groups.

**Conclusion:**

The FOPR test was valid for the HSN diagnosis and provided quantitative and intuitive information regarding visuospatial function. Furthermore, it might enhance our understanding of visuospatial function including HSN by applying the time relative component and concepts of perception and exploration, FOP and FOR.

*Trial registration*: NCT03463122. Registered 13 March 2018, retrospectively registered.

## Background

Hemispatial neglect (HSN) manifests as slow, incomplete, or absent responses to stimuli presented in the area of the visual field contralateral to the injured hemisphere [1, 2]. HSN is common in stroke patients and is considered a poor prognostic factor [3–5]. Many different brain lesions have been implicated in HSN, including those of the right cortical area (e.g. temporo-parietal junction, inferior and middle frontal gyri, inferior parietal lobe) and the right subcortical area (basal ganglia, or thalamus) [6]. Recently, white matter lesion or lesion of intra and inter-hemispheric connections have been associated with HSN [7, 8]. Similarly, various pathophysiologic mechanisms, including perception-attention, representation of space, and motor-intentional aiming, could be linked to HSN [9].

These multiple lesions and mechanisms make diagnosis of HSN difficult, and various approaches have been applied to assess HSN. Paper-and-pencil tests, such as line bisection, cancellation, drawing, figure copying, and reading, have been widely used [10, 11]. These tests help to diagnose HSN symptoms; however, a systematic review suggested that they have low diagnostic value with low sensitivity and specificity, since they cannot quantify HSN and scoring is only performed with regard to the horizontal axis [12]. Moreover, patients become accustomed to these tests and easily compensate for their HSN. Recently, functional assessments, such as the Catherine Bergego scale (CBS), have been proposed for HSN evaluation [10]. However, they are hindered by evaluator dependency and their administration is time-consuming.

Virtual reality (VR) approaches with computer-based digitalised evaluation and accurate sensors were found to have higher sensitivity and specificity and better performance for HSN detection when compared with classic paper-and-pencil tests [13–15]. VR allows humans to interact with computer-generated realistic environments, which enables researchers to control stimuli presentation and to obtain objective and detailed measurements of performance.

In a previous study, we proposed a novel VR-based method to assess binocular visual stimuli detection, herein referred to as the FOPR test [16]. The FOPR test was based on the notion that the human eye has a limited visual field; thus, the full exploration of a visual scene requires movement of head and body. Therefore, the human visual search pattern can be differentiated into field of perception (FOP) and field of regard (FOR) depending on the head and body movement. Specifically, FOP refers to the size/angle of the visual field that is visible at any given moment without head and body movement, whereas FOR refers to the total range of the visual field in the real world, which involves moving the head or body to view the surroundings [16, 17].

Classic paper-and-pencil tests cannot distinguish between FOP and FOR as they do not measure posture and head or trunk movements restraints during evaluation. Hence, their contribution to HSN has yet to be explored. However, a head-mounted display (HMD) allows FOPR test to evaluate both FOP and FOR separately. FOP is not affected by head rotations or body movements because the HMD is fixed on the participant’s head and the screen does not change with movements. However, FOR requires exploration involving head and body movements. In this condition, when the participant performs head movements, computer-generated images move as if the participant is in the real world. We believe that human FOP and FOR represent perception and exploration, respectively, and enable the classification of HSN into perceptual and exploratory neglect [18]. Moreover, unlike conventional tests, FOPR test enables quantification of visuospatial function assessments using a time-related factor, leading to highly sensitive measurements of HSN.

Therefore, we hypothesized that the FOPR test could enable novel measurements of visuospatial function to assess HSN. The purpose of our study was to ensure the validity and accuracy of the FOPR test. Initially, we explored the diagnostic validity of the FOPR test for HSN using Bland–Altman analysis and Support vector machine (SVM) classification. Lastly, we compared the results of the FOPR test between three groups (stroke patients with a lesion in the right hemisphere and with HSN [HSN+SS group], stroke patients with a lesion in the right hemisphere and without HSN [HSN−SS group] and healthy controls [HC group]).

## Methods

### Participants

The study included right-handed participants aged 19–65 years. Participants with oculomotor palsies, visual field defects, or cognitive impairments (score ≤ 25 in the Mini-Mental State Examination) were excluded [19]. Additionally, participants with orthopaedic disorders affecting neck or trunk movement, those who could not sit for approximately 10 min, and those with right upper limb use difficulty that impeded their ability to click the mouse were excluded because of their inability to perform the FOPR test. Participants with stroke were included if they experienced first-ever right hemispheric stroke resulting in left hemiplegia at least 3 months ago, as evidenced by brain imaging and medical records. The presence of HSN was determined using the conventional tests described in the ‘Conventional HSN tests’ section below.

Among 73 stroke patients consecutively admitted to a rehabilitation hospital, we enrolled 19 participants in the HSN+SS group. Only patients with left-sided HSN corresponding to their right hemispheric lesion were included in the HSN+SS group. Subsequently, we consecutively enrolled 22 age-matched participants in the HSN−SS group and 22 in the HC group. Demographic characteristics of the participants are presented in Table 1. The stroke patients were recruited from one rehabilitation hospital by a physiatrist who manages the neurorehabilitation department. Meanwhile, part of the HC group was recruited by a co-authoring physiatrist, J. Y. L, from the rehabilitation hospital, and part by a co-authoring researcher, C. Y. C, from a university using notice board postings in both cases. Medical doctors checked the inclusion and exclusion criteria, and one research occupational therapist performed the experiment including the HSN assessments at the rehabilitation hospital between December 2014 and February 2018. This study was approved by the institutional review boards of the rehabilitation hospital and university, and all participants provided written informed consent prior to enrolment. This study was registered at clinicaltrials.gov (NCT03463122).

**Table 1:** Demographic and baseline clinical characteristics

	HSN+SS (n = 19)	HSN−SS (n = 22)	HC (n = 22)	p-value
Age, y	54.32 ± 7.40	49.23 ± 9.99	45.41 ± 17.82	0.094
Male sex, n (%)	14 (73.68)	17 (77.27)	10 (45.45)	0.055
Months after stroke	11.79 ± 12.37	10.57 ± 10.96	–	0.749
Conventional tests				
Line bisection test	5.79 ± 3.00	8.91 ± 0.29	–	< .001
Star cancellation test	45.37 ± 9.34	53.73 ± 0.77	–	0.001
Catherine Bergego scale	6.16 ± 4.59	0.18 ± 0.85	–	< .001

One-way ANOVA was used for age; chi-square test was used for sex; independent t-test was used for months following stroke and conventional tests. Values are shown as means ± standard deviations

*HSN+SS* stroke patients with a lesion in the right hemisphere and with HSN, *HSN−SS* stroke patients with a lesion in the right hemisphere and without HSN

### Conventional HSN tests

All participants with stroke (HSN+SS and HSN−SS groups) were assessed for HSN symptoms using the line bisection test (LBT) and star cancellation test (SCT) of the Behavioural Inattention Test and CBS [10, 11, 20]. The presence of HSN was determined according to the following criteria: (1) an LBT score of < 7, (2) an SCT score of < 51, or (3) presence of neglect-related functional impairment defined by a CBS score of > 1. Since there are no established golden standard criteria for HSN, this combination of tests has been shown to improve the reliability of its diagnosis [21].

Both the LBT and SCT were performed using an A4 sheet of paper presented in front of each participant’s mid-sagittal line. Participants in the HC group did not undergo these conventional tests. In the LBT, participants were presented with a sheet of paper containing three horizontal lines depicted like a staircase and were asked to bisect the lines by marking the centre of each line using their preferred or unaffected hand. The deviation of the marking from the true centre was then converted to a score that ranged from 0 to 3, and the total score ranged from 0 to 9. In the SCT, participants were presented with a sheet of paper containing 56 small stars interspersed among distractors and were asked to mark the small stars [11]. The total number of marked small stars was considered as the score, ranging from 0 to 54 (subtracting two stars for demonstration). In both the LBT and SCT, lower scores reflected more severe HSN.

In the CBS assessment, a standardised checklist designed to detect the degree of HSN during everyday life was composed by a research occupational therapist via direct observation of functioning during tasks [20]. The score for each task ranged from 0 to 3, and the total score ranged from 0 to 30. In this assessment, high scores indicated more severe HSN.

### FOPR test

The FOPR test was performed using a stereo HMD system (Oculus Rift DK1, Oculus VR, Irvine, CA, USA) and a three-dimensional development platform (Vizard 4.0; WorldViz, Santa Barbara, CA, USA) controlled by a desktop workstation running Windows 7 (Microsoft, Redmond, WA, USA) that was equipped with a high-end graphics card (NVIDIA GTX 760Ti; NVIDIA, Santa Clara, CA, USA). The screen resolution was 1280 × 800 (640 × 800 per eye), and a built-in three-degrees-of-freedom sensor was used to track head movements. When the sensor is turned off and on, evaluations of FOP and FOR are possible, respectively.

A white fixation cross was displayed at the centre of the screen before each trial. During the FOP condition, a research occupational therapist instructed the participants to constantly look at the white fixation cross between each trial. As the white cross disappeared, the target was presented at the same time. During the FOR condition, the HMD centre, identified by the embedded sensor, was displayed as a red cross on the VR. Before each trial, the patients were asked to move their heads so that the red cross was aligned with the white fixation cross. Once this occurs, the white fixation cross would disappear, and the target would be presented. The targets where either red or blue spheres that were presented at predefined locations in the VR environment (see FOP measurement). The location of the target was chosen randomly in each trial. Both colours appeared in equal frequency and were equally presented on the right and left sides. Participants were instructed to click the left or right button of a computer mouse as fast as possible when they saw a blue or red target, respectively. After a click, the target immediately disappeared. If participants failed to find the target or did not click a button within a time limit (FOP measurement: 5 s, FOR measurement: 10 s), the test automatically proceeded to the next trial. After each trial, auditory feedback for correct and wrong responses was provided. The inter-trial interval varied between 0.5 and 1.5 s, and the FOPR test took about 10–20 min to accomplish.

### FOP measurement

**Fig. 1:**
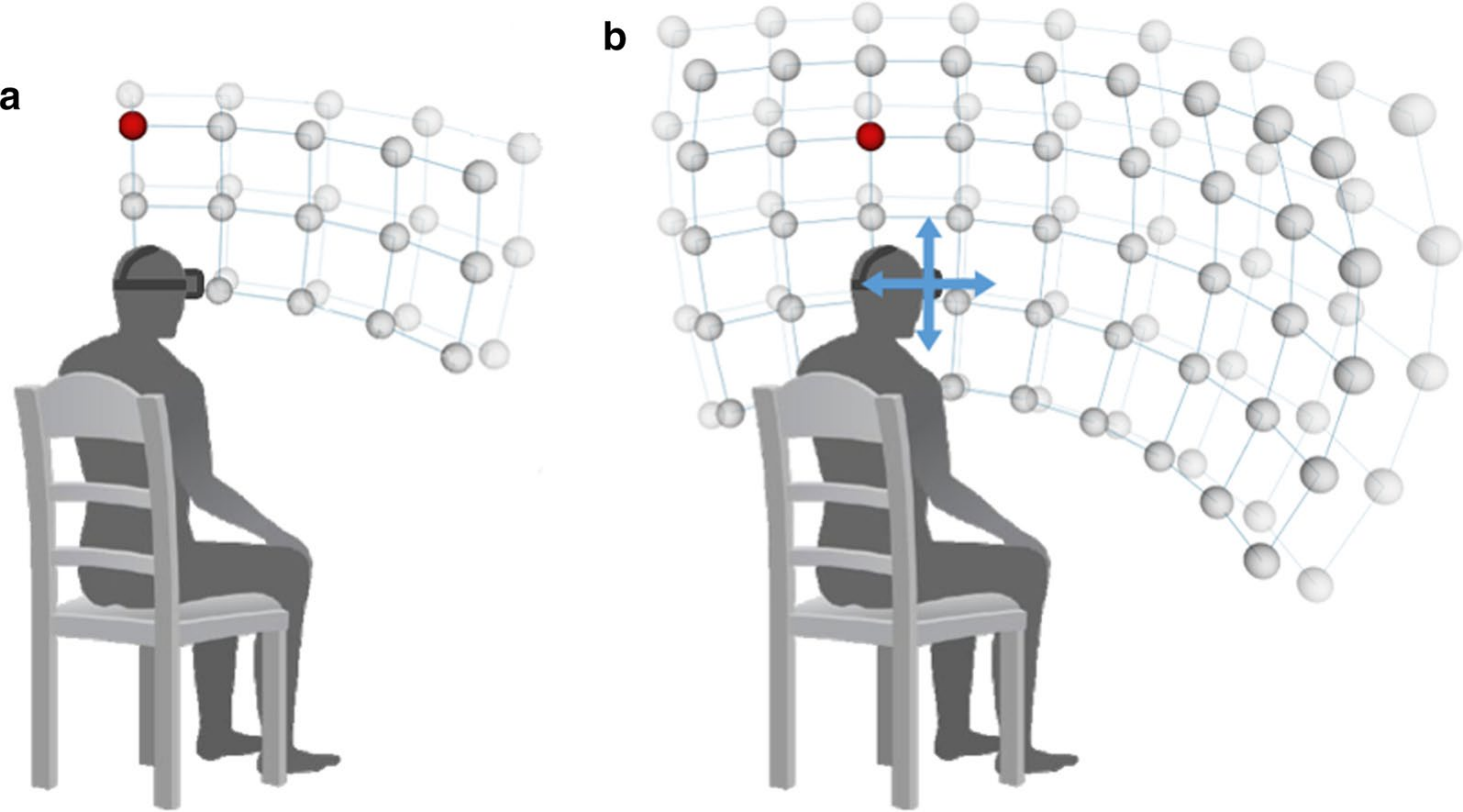
Arrangement of stimuli in the spherical coordinate system. **a** FOP condition; **b** FOR condition. FOP, field of perception; FOR, field of regard

The FOP measurement assesses the ability of a participant to detect a target in the absence of head movement. To obtain a measurement for FOP, the head-tracking feature of the HMD was turned off to ensure that the participant’s view of the screen remained constant regardless of head movement. The FOP measurement was obtained from 30 trials where 30 targets were randomly presented on a 5 × 3 × 2 (horizontal × vertical × radial) spherical coordinate system (Fig. 1a; horizontal: 60°, vertical: 30°; near–far positions in the radial direction) within the FOP of the experimental equipment (Oculus; horizontal: 93.3°, vertical: 58.3°).

### FOR measurement

For the FOR measurement, the head-tracking feature of the HMD was turned on to ensure that the participant’s view of the screen changed in accordance with head rotation. Participants were asked to move the head to detect a target as quickly as possible. The FOR measurement was obtained from 90 trials where 90 targets were randomly presented on a 9 × 5 × 2 (horizontal × vertical × radial) spherical coordinate system (Fig. 1b; horizontal: 120°, vertical: 60°; near–far positions in the radial direction). Each target in the FOPR test was located at an interval distance of 15° from the centre of the screen.

### FOPR variables

The FOPR variables were success rate (SR; FOPR-SR), which was defined as the percentage of correct responses (clicking the left or right mouse button in response to a blue or red target, respectively within time limit); and response time (RT; FOPR-RT), which was defined as the time interval between target appearance and mouse click. Therefore, the FOPR variables included FOP-SR and FOP-RT for FOP, and FOR-SR and FOR-RT for FOR. The RTs of failed trials were replaced with the maximum time limit, which was 5 s and 10 s for FOP-RT and FOR-RT, respectively. For further analysis, FOP-RT and FOR-RT were calculated separately for the left and right spaces (specified by adding left or right at the variable, e.g., FOP-RT-Right for FOP-RT in the right space) excluding midline trials.

### Paired t-test and Spearman correlation analysis

The purpose of the study was to analyse the HSN in two dimensions, therefore, we tried combining two radial planes with analytic basis. We verified the values using paired t-test and Spearman correlation analysis, and although there was strong correlation between two planes, there was a significant difference between near and far planes. Thus, we separated the values according to radial axis by adding Near or Far at the end of the variables, e.g., FOP-SR-Near for FOP-SR in the near plane, and conducted analysis in each radial plane.

### Bland–Altman analysis

In order to confirm the validity of the FOPR test, Bland–Altman analysis was performed to assess the agreement between the z-score of the conventional tests and that of the FOPR variables among the HSN+SS group [22]. The mean difference between z-scores of FOPR variables and conventional tests was set to zero since the values of tests were standardised into z-scores. Limits of agreements were obtained at the mean ± 1.96 standard deviation. For the 95% confidence interval, we had 18 degrees of freedom and t distribution = 2.101. Accordingly, confidence intervals were obtained as the mean ± 2.101 standard error.

### Support vector machine (SVM) classification

Combinations of SR and RT for each of FOP and FOR (FOP-SR&RT, FOR-SR&RT) were fed to a SVM classification algorithm [23]. The data set consisted of FOP-SR&RT or FOR-SR&RT of the right, left, and entire space from the near and far planes. For example, we utilized two data points (FOP-Left-Near-SR and FOP-Left-Near-RT), when we used the FOP-Left-Near variables to distinguish between the two groups (HSN vs non-HSN groups). The SVM classifications were conducted using mapping features onto a multidimensional space and constructing hyperplanes, which maximised the margin between observations of different groups. The SVM classifiers were configured using C-classification with the radial basis kernel function and fivefold cross validation. Thus, we trained 80% of the participants (51 subjects) and tested classifier performance using the remaining 20% (12 subjects). Because the units of the two variables (SR and RT) were different, the value ranges were normalized to range between 0 and 1, in order to eliminate any bias from the differences in the units. The hyperparameters of the SVM, γ and C, were tuned using a grid search over all reasonable parameters, in which γ ranged from 0.0001 to 10 and C ranged from 1 to 1000 [24]. SVM classifications were performed with R package e1071 and its interface with LIBSVM [25, 26].

We used FOP-SR&RT and FOR-SR&RT of the right and left space for the SVM classification in order to define whether those variables on each hemispace belong to different groups. Thus, we performed SVM analyses to define whether FOP-SR&RT or FOR-SR&RT in the left hemispace of HSN were different from those of the non-HSN. We repeated this procedure for the right hemispace. We determined whether the FOPR test can be applied to diagnosing HSN, if the SVM correctly classifies the groups for the compromised left hemispace, but misclassifies the ones related to the non-compromised right hemispace. SVM classification using the FOPR variables from both spaces was also carried out for the references. We performed these processes in the near and far space, respectively. Therefore, SVM classifications for binary-classifier (HSN vs non-HSN) was conducted to determine the validity of FOPR test for HSN detection. Then, we evaluated the classifier’s performance using the confusion matrix generating classification accuracy (the number of correctly classified observations per total observations), sensitivity, specificity, as well as positive predictive value (PPV), and negative predictive value (NPV). Therefore, the confusion matrix indicates the proportion of correct classification of the test data of 12 subjects.

### Statistical analysis

One-way analysis of variance (ANOVA) or Kruskall–Wallis tests for continuous variables (depending on normality according to Kolmogorov–Smirnov test) and the chi-square tests for categorical variables were used for comparisons across the three groups. Post-hoc comparisons were performed with Bonferroni correction in case of a significant between-group difference. In addition, comparisons between two groups were performed using the t-test for continuous variables. All statistical analyses were performed using R 3.6.2 (http://www.r-project.org), and statistical significance was set at a p-value < 0.05 [27].

## Results

### Demographic characteristics and conventional test results

Demographic characteristics of the participants and conventional test results are presented in Table 1. There were no significant differences in age, sex, and time from onset of stroke across the groups. Conventional test results were significantly different between the HSN+SS and HSN−SS groups.

### Diagnostic validity of the FOPR test for HSN

#### Agreement between the FOPR test and conventional tests

**Fig. 2:**
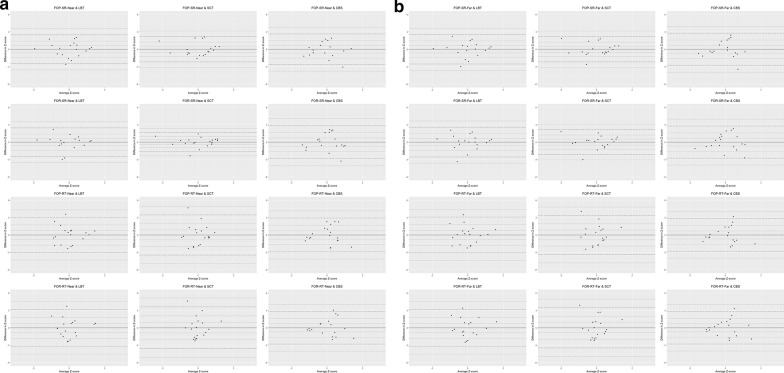
Bland–Altman analysis plots. **a** Variables of the near plane are on the left side and **b** those from the far plane are on the right side. Mean difference (bold line), limits of agreement for the mean difference (dashed lines) and 95% confidence interval of limits of agreement (dotted lines) for the z-scores of FOPR variables (FOP-SR, FOR-SR, FOP-RT, and FOR-RT) and conventional tests (LBT, SCT, and CBS) among patients with HSN. *FOP* field of perception; *FOR* field of regard; *SR* success rate; *RT* response time; *HSN* hemispatial neglect; *LBT* line bisection test; *SCT* star cancellation test; *CBS* Catherine Bergego scale

Figure 2 presents Bland–Altman plots of the z-scores of the FOPR variables and the conventional tests. Bland–Altman plots of the HSN+SS group showed a random fluctuation around the mean. In the Bland–Altman plot between FOPR variables and conventional tests, 5.3% of individuals were outside the limits of agreement, except between LBT and FOR-SR-Near (10.5%), CBS and FOR-RT-Near (0%), and CBS and FOR-SR (0%). Additionally, all subjects were within the 95% limits of agreement except for one each between SCT and FOR-SR-Near and between SCT and FOR-SR-Far.

#### Classification using support vector machine

**Fig. 3:**
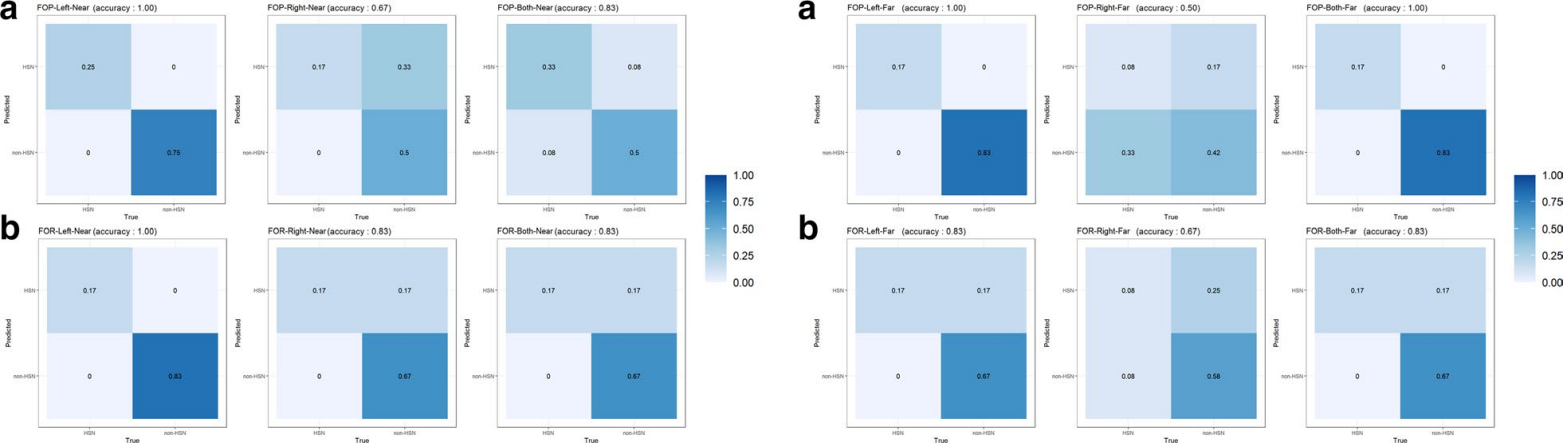
Confusion matrix for the binary support vector machine classifier. Numbers correspond to the ratio of the time the classifier chose that trial type. Confusion matrix from the near plane are on the left side and from the far plane on the right side. Graphs of **a** FOP and **b** FOR are displayed in the order of the Left (left), Right (middle) and Both (right) hemispace (s)

The confusion matrices of the SVM classifications for the binary-classifier (HSN vs. non-HSN) are shown in Fig. 3. The accuracy of the SVM classification varied from 50.0 to 100% for the binary-classifier. FOP-SR&RT of the left hemispace had a 100% accuracy, while that of right hemispace had a 50.0% or 66.7% accuracy. Similarly, FOR-SR&RT of the left hemispace showed 100% or 83.3% of accuracy, while that of the right hemispace showed 83.3% or 66.7% accuracy. As for the comparisons of FOR and FOP, FOP-SR&RT showed higher or equal accuracies than FOR-SR&RT in the left hemispace, and FOR-SR&RT showed higher accuracies than FOP-SR&RT in the right hemispace. The accuracies of the SVM classification using FOP-RT&SR or FOR-RT&SR of both spaces exceeded 83.3%.

#### FOPR test results

Differences of FOPR variables among the groups are shown in Table 2. FOPR-SRs were significantly different between groups (all, p < 0.05) except FOR-SR-Right-Near and FOP-SR-Right-Far (p-value = 0.075 and 0.452, respectively), and post-hoc analysis revealed that HSN+SS group had significantly lower SR than the other groups. However, there was no significant difference between HSN−SS and HC groups in all the FOPR-SRs.

**Table 2:** Comparison of FOPR-SR

	Mean rank			χ2	p-value	Post-hoc
	HSN+SS	HSN−SS	HC			
FOPR-SRs from near plane						
FOP-SR-Near	15.37	41.30	37.07	24.64	< 0.001	HSN+SS < HSN−SS, HC
FOP-SR-Left-Near	14.84	41.18	37.64	29.46	< 0.001	HSN+SS < HSN−SS, HC
FOP-SR-Right-Near	22.89	36.59	35.27	9.04	0.01	HSN+SS < HSN−SS, HC
FOR-SR-Near	13.82	40.93	38.77	28.92	< 0.001	HSN+SS < HSN−SS, HC
FOR-SR-Left-Near	13.63	39.36	40.50	32.08	< 0.001	HSN+SS < HSN−SS, HC
FOR-SR-Right-Near	25.58	41.86	33.45	5.18	0.075	
FOPR-SRs from far plane						
FOP-SR-Far	12.45	41.07	39.82	31.99	< 0.001	HSN+SS < HSN−SS, HC
FOP-SR-Left-Far	11.82	40.55	40.89	35.80	< 0.001	HSN+SS < HSN−SS, HC
FOP-SR-Right-Far	22.89	35.20	31.45	1.59	0.452	
FOR-SR-Far	12.76	40.93	38.77	32.17	< 0.001	HSN+SS < HSN−SS, HC
FOR-SR-Left-Far	12.24	40.66	40.41	35.78	< 0.001	HSN+SS < HSN−SS, HC
FOR-SR-Right-Far	18.89	41.86	33.45	18.33	< 0.001	HSN+SS < HSN−SS, HC

Kruskall–Wallis tests and Mann–Whitney U-test for comparisons of success rate (SR) between groups and post-hoc study

*FOPR* FOP and FOR, *FOP* field of perception, *FOR* field of regard, *HSN+SS* stroke patients with a lesion in the right hemisphere and with HSN, *HSN−SS* stroke patients with a lesion in the right hemisphere and without HSN, *HC* healthy control

FOPR-RTs all showed significant difference between groups (all, p < 0.001) (Table 3) and post-hoc analysis revealed that HSN+SS group had significantly longer RT than the other groups except FOP-RT-Right-Far, in which there was no difference between HSN+SS and HSN−SS (p = 0.136). Significant differences between HSN−SS and HC were found in the FOR-RT, FOR-RT-Left in both radial spaces, and FOR-RT-Right-Far; HSN−SS had longer RT than HC.

**Table 3:** Comparison of FOPR-RT

	Mean ± SD			Mean square(df)		F-value	p-value	Post-hoc
	HSN+SS	HSN−SS	HC	Between groups (2)	Within groups (60)			
FOPR-RTs from near plane								
FOP-RT-Near	1.83 ± 0.73	0.83 ± 0.16	0.59 ± 0.14	8.60	0.18	48.49	< 0.001	HC < HSN−SS, HSN+SS
FOP-RT-Left-Near	2.86 ± 1.63	0.89 ± 0.79	0.59 ± 0.15	30.41	0.60	51.09	< 0.001	HC < HSN−SS, HSN+SS
FOP-RT-Right-Near	1.07 ± 0.45	0.79 ± 0.22	0.59 ± 0.15	1.19	0.09	13.80	< 0.001	HC < HSN−SS, HSN+SS
FOR-RT-Near	4.34 ± 1.58	2.19 ± 0.58	1.30 ± 0.44	49.12	0.93	52.61	< 0.001	HC < HSN−SS < HSN+SS
FOR-RT-Left-Near	6.04 ± 2.22	2.55 ± 0.82	1.29 ± 0.43	121.40	1.78	68.52	< 0.001	HC < HSN−SS < HSN+SS
FOR-RT-Right-Near	3.14 ± 1.53	2.06 ± 0.58	1.42 ± 0.51	15.29	0.91	16.86	< 0.001	HC < HSN−SS, HSN+SS
FOPR-RTs from far plane								
FOP-RT-Far	2.10 ± 0.69	0.96 ± 0.22	0.65 ± 0.18	11.67	0.17	68.86	< 0.001	HC < HSN−SS < HSN+SS
FOP-RT-Left-Far	3.34 ± 1.18	1.06 ± 0.33	0.64 ± 0.17	42.11	0.47	90.18	< 0.001	HC < HSN−SS, HSN+SS
FOP-RT-Right-Far	1.20 ± 0.60	0.95 ± 0.30	0.68 ± 0.23	1.39	0.16	8.78	< 0.001	HC < HSN+SS
FOR-RT-Far	5.03 ± 1.63	2.46 ± 0.73	1.39 ± 0.50	70.41	1.07	65.55	< 0.001	HC < HSN−SS < HSN+SS
FOR-RT-Left-Far	6.61 ± 1.86	2.71 ± 1.02	1.30 ± 0.45	151.51	1.47	102.84	< 0.001	HC < HSN−SS < HSN+SS
FOR-RT-Right-Far	3.86 ± 1.74	2.42 ± 0.67	1.58 ± 0.61	26.81	1.20	22.40	< 0.001	HC < HSN−SS < HSN+SS

One-way analysis of variance (ANOVA) and Bonferroni correction for comparisons of reaction time (RT) between groups

*FOPR* FOP and FOR, *FOP* field of perception, *FOR* field of regard, *HSN+SS* stroke patients with a lesion in the right hemisphere and with HSN, *HSN−SS* stroke patients with a lesion in the right hemisphere and without HSN, *HC* healthy control

### FOPR-RT heat map

**Fig. 4:**
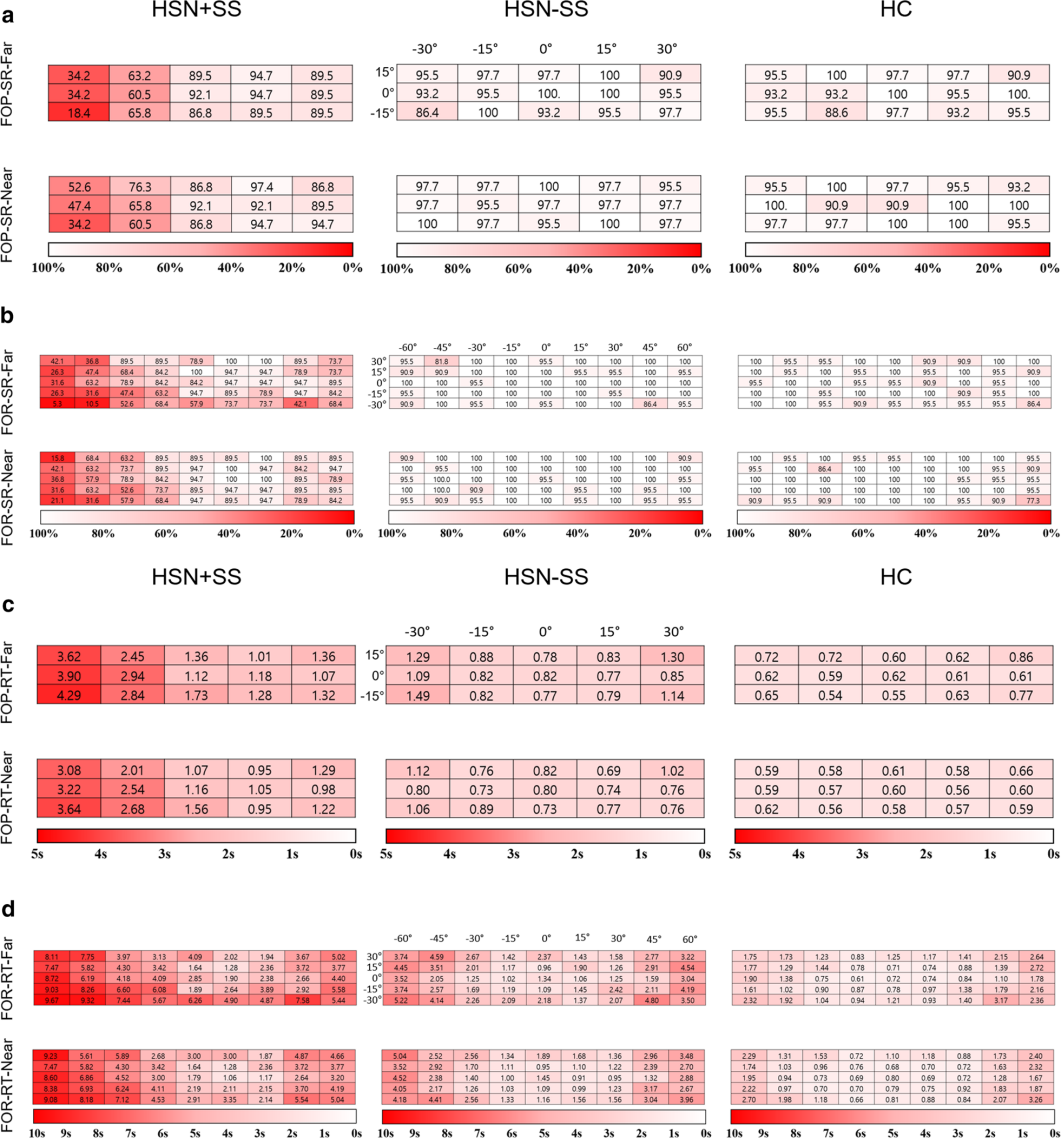
Heat map using the mean values of each group. Variables of far plane are at the top and from near plane at the bottom for all panels. Heat maps of **a** FOP-SR, **b** FOR-SR, **c** FOP-RT and **d** FOR-RT are displayed in the order of HSN+SS (left), HSN−SS (middle) and HC (right). SR close to 100% and RT close to 0 s, as shown in white, and the opposite maximum values, as shown in red. Each column and row represent horizontal and vertical axis, respectively and the exact angle of each axis is shown at the middle figures of far plane. *FOP* field of perception; *FOR* field of regard; *SR* success rate; *RT* response time; *HSN* hemispatial neglect; *HSN+SS* stroke patients with a lesion in the right hemisphere and with HSN; *HSN−SS* stroke patients with a lesion in the right hemisphere and without HSN

We generated heat maps for each group using the mean value of FOPR variables (Fig. 4) to show the overview across groups. We used sequential colour schemes in which SRs close to 100% and RTs close to 0 s are shown in white, and those close to 0% SR and maximum time limit are shown in red. Each column and row in the heat map presented each target point in a horizontal and vertical axis, respectively. Overall, the three groups commonly demonstrated the lowest RT values in the central area of FOP and FOR. The higher values at both ends were more evident of FOR heat map than FOP, suggesting more demands on head rotation of FOR than of FOP. The sequential pattern at both ends was more evident with HSN+SS group followed by HSN−SS and HC groups, especially at the left side.

## Discussion

### Diagnostic validity of the FOPR test for HSN

We demonstrated that FOPR test might be a valid tool for assessment of the visuospatial function, including HSN based on agreement and SVM classification accuracy. First, we found that the FOPR test was valid for HSN diagnosis, according to its agreement with conventional HSN tests. The Bland–Altman plots showed the FOPR variables had good agreement with conventional tests, ranging from 89.5 to 100%, although floor or ceiling effects of conventional tests may have contributed to the variance of the agreements. Additionally, good agreement of FOPR test with CBS indicated that FOPR test could reflect the functional aspect of HSN. In particular, consistent good agreement of various FOPR variables including SR and RT of FOP and FOR showed that these variables can play a role in HSN assessment.

While most conventional HSN tests have measured SR, but not time-associated variables, diagnostic tools using RT have been recently used to identify HSN more accurately. [28–30] RT could indicate degradation of attention on the contralateral side and also subtle changes or differences [31, 32]. Importantly, in the FOPR test, both RT and SR were obtained simultaneously and were not time-consuming. Thus, it could be easily applied in the clinical settings.

Subsequently, we demonstrated that FOPR test combining SR and RT could classify HSN. SVM classification of the FOPR test achieved 100% accuracy using FOP-SR&RT from the left hemispace; however, using variables from the right hemispace showed poor classification. It demonstrated reliable performance of the FOPR test for HSN assessment because the visuospatial deficits of HSN occurred mainly in the left space. In addition, the reason for 100% accuracy of the classification using FOP variable might be because HSN was mainly determined with conventional test using an A4 sheet. Thus, no head movement was required, thereby mimicking FOP.

Similar to the classification using FOP-SR&RT, the accuracies of the binary classification using FOR-SR&RT in the left hemispace (83.3% or 100%) were higher than those of the FOR-SR&RT in the right hemispace, indicating the potential for FOR-SR&RT to classify the HSN. To sum up, confusion matrices indicated that the classification accuracy from the FOP-SR&RT and FOR-SR&RT of the left hemispace or both spaces exceeded 83.3%. In addition, SVM binary classification for HSN results showed high sensitivity and PPV (see Additional file 1). This confirms the robustness of the HSN classification using FOPR variables. These findings indicate that FOPR tests, in which subclassified measurements on both FOP and FOR and the time-relevant characteristics of FOPR-RT, which were not incorporated in the conventional tests, might have contributed to visuospatial assessment performances.

### FOPR variables across groups

The FOPR test demonstrated differences across groups, consistent with conventional tests and SVM classification. SR is significantly different between HSN+SS group and the other groups when analysed across both sides and left space only, suggesting that values from entire and left space are optimized for simple diagnosis of HSN demonstrating clarity during diagnosis. Some of the variables from the right side, FOP-SR-Right-Far and FOR-SR-Right-Near, could not even confirm the difference between groups unlike the other space and its consistency with HSN characteristics of our participants.

However, all variables regarding RT showed between-group differences, and all RT variables except FOP-RT-Right-Far showed differences between the HC group and the other groups. This finding indicates that the SR was different in HSN+SS based on HSN, while the RT showed a difference in HC compared to subjects with stroke. This difference is consistent with a previous study, in which stroke patients showed delayed RT to a visual stimulus compared to healthy controls [33]. Interestingly, post-hoc analyses demonstrated that most of the FOR-RT variables (FOR-RT-Near, FOR-RT-left-Near, FOR-RT-Far, FOR-RT-Left-Far, FOR-RT-right-Far) showed significant differences and uniform pattern across the three groups (HC < HSN−SS < HSN+SS group), whereas only one FOP-RT variable (FOP-RT-Far) showed differences across three groups. Considered together, SR and RT, and FOP and FOR might have a divergent role for visuospatial function measurements. Overall, these results indicated that FOPR test might be a valid tool for visuospatial function assessment, including HSN.

In addition to this assessment performance, evaluation of both FOP and FOR allows HSN subtyping. HSN could be subclassified according to perceptual-attentional and motor-intentional deficits [34]. Similarly, the FOPR test could explore whether the HSN is attributed to relative sparing or impairment of FOP or FOR, which is considered to be related to perception-attention or motor-intention, respectively because the FOR requires exploration via head rotation and is thus more challenging than FOP. Moreover, this classification approach could enable personalised HSN rehabilitation. For example, exploration training emphasizing head rotation could be recommended for HSN patients with FOR impairment, rather than for HSN patients without FOR impairment, similar to a previous study [34].

Additionally, using the heat map, we can appreciate the above results with just a glance. Moreover, the heat map revealed more detailed image of visuospatial function among groups. This kind of mapping will help both clinicians and patients gain a more intuitive understanding of the current status and better track treatment progress.

Although visuospatial function depending on the radial axis was not our original concern, we found that the RT values of the near plane were shorter than those of the far plane in the same condition as seen in Table 3. This suggested that stereopsis was preserved with stroke patients, even for HSN+SS. Furthermore, the post-hoc significant difference between HSN+SS and HSN−SS was found in four variables from the far plane (FOP-RT-Far, FOR-RT-Far, FOR-RT-Left-Far, FOR-RT-Right-Far) and one variable (FOP-RT-Near) from the near plane. Thus, the variables of the far plane might detect subtle visuospatial function differences between HSN+SS and HSN−SS. Therefore, a target located far away might be more useful for VR, such as a FOPR test or visuospatial function test, in order to detect subtle difference in visuospatial function.

The FOPR test has some merits when compared with conventional tests. First, it differentiates visuospatial function into FOP and FOR, providing a more detailed picture of visuospatial function. Clinically, this knowledge could help identify the optimal treatment strategy according to a patient's visual perceptual and exploratory function.

Second, the FOPR test allows highly sensitive quantification of visuospatial function, using RT as well as SR. Therefore, the FOPR test could be used to detect mild visuospatial functional decline that cannot be detected in any of the conventional tests and lead to proper rehabilitation. Third, the FOPR test allows highly reproducible quantified recording of all trials. Conventional tests have no detailed instructions and can be easily affected by the evaluator or environment. The FOPR test can overcome these shortcomings and allow evaluator-independent, automatic evaluation within a uniform environment in a computer setting. Fourth, The FOPR test has practical significance because it can be used in a wide range of participants since it only requires the ability to sit and click a mouse button, making it appropriate in a clinical environment. Moreover, the affordable price of the HMD system may make the FOPR test more widely available.

This study has several limitations. First, we did not limit or measure eye movement during the FOPR test; therefore, we measured only the field of perception instead of the field of view. Eye-tracking systems are needed to capture a field of view suggesting pure perceptual function. Second, FOR measures comprised three times the number of trials than FOP measures because of the larger FOR spherical coordinate space, resulting in different weights of values between FOP and FOR. Third, the FOPR test presents only one trial at each coordinate in three dimensions. More trials for each coordinate would strengthen the FOPR test. Fourth, we did not investigate the visuospatial function on the radial axis. Although there were differences in FOPR variables between near and far planes, there were no consistent findings. Additionally, because the main purpose of this study was to validate the FOPR test on two dimensions, we did not measure the radial plane further. If we analysed the visuospatial function according to the radial axis, it could be possible to further differentiate HSN subtypes according to space (personal, near extrapersonal, and far extrapersonal spaces) [35]. Fifth, the FOPR test did not address functional tasks, such as activities of daily living, as we attempted to focus on neurophysiological characteristics in a controlled experimental setting. Further studies involving functional everyday tasks in a real environment could be more meaningful and easily linked to interventions. Sixth, the study did not include stroke patients with left hemispheric lesions or those in the acute phase. Thus, our results cannot be generalised.

## Conclusion

Our VR-based visuospatial assessment tool, the FOPR test, was valid for HSN diagnosis, showed high agreement with a combination of conventional tests, and proved to have a high accuracy in diagnosing HSN. It may provide highly elaborate tools for HSN, in addition to quantitative and comprehensive information regarding visuospatial function in terms of FOP and FOR, allowing for the use of novel indices.

## Supplementary Information

**Additional file 1:Table 1.** The performance of SVM accuracy, sensitivity, and specificity for binary-classifier (HSN vs non-HSN).

## Data Availability

The datasets used and/or analysed during the current study are available from the corresponding author on reasonable request.

## Abbreviations

CBS: Catherine Bergego Scale
FOP: Field of perception
FOR: Field of regard
HC: Healthy control
HMD: Head-mounted display
HSN: Hemispatial neglect
HSN+SS: Stroke patients with a lesion in the right hemisphere and with HSN
HSN−SS: Stroke patients with a lesion in the right hemisphere and without HSN
LBT: Line bisection test
RT: Response time
SCT: Star cancellation test
SR: Success rate
SVM: Support vector machine
VR: Virtual reality
