# Supplementary material for: FOPR test: a virtual reality-based technique to assess field of perception and field of regard in hemispatial neglect
Source: J Neuroeng Rehabil. 2021 Feb 18;18:39. doi: 10.1186/s12984-021-00835-1 (PMC7890954; doi:10.1186/s12984-021-00835-1)
Supplement: Supplementary file 1 — Additional file 1:Table 1. The performance of SVM accuracy, sensitivity, and specificity for binary-classifier (HSN vs non-HSN). [file 12984_2021_835_MOESM1_ESM.docx]

Additional Table 1. Performance of SVM accuracy, sensitivity, and specificity for binary-classifier (HSN vs non-HSN)

| SVM classifier | Accuracy | HSN sensitivity | HSN specificity | HSN PPV | HSN NPV |
| --- | --- | --- | --- | --- | --- |
| FOPR variables from the near plane | | | | | |
| FOP-SR&RT-Left-Near | 100.0% | 100.0% | 100.0% | 100.0% | 100.0% |
| FOP-SR&RT-Right-Near | 66.7% | 100.0% | 60.0% | 33.3% | 100.0% |
| FOP-SR&RT-Both-Near | 83.3% | 80.0% | 85.7% | 80.0% | 85.7% |
| FOR-SR&RT-Left-Near | 100.0% | 100.0% | 100.0% | 100.0% | 100.0% |
| FOR-SR&RT-Right-Near | 83.3% | 100.0% | 80.0% | 50.0% | 100.0% |
| FOR-SR&RT-Both-Near | 83.3% | 100.0% | 100.0% | 50.0% | 100.0% |
| FOPR variables from the far plane | | | | | |
| FOP-SR&RT-Left-Far | 100.0% | 100.0% | 100.0% | 100.0% | 100.0% |
| FOP-SR&RT-Right-Far | 50.0% | 20.0% | 71.4% | 33.3% | 55.6% |
| FOP-SR&RT-Both-Far | 100.0% | 100.0% | 100.0% | 100.0% | 100.0% |
| FOR-SR&RT-Left-Far | 83.3% | 100.0% | 80.0% | 100.0% | 50.0% |
| FOR-SR&RT-Right-Far | 66.7% | 50.0% | 70.0% | 25.0% | 87.5% |
| FOR-SR&RT-Both-Far | 83.3% | 100.0% | 80.0% | 50.0% | 100.0% |

SVM, support vector machine; HSN, hemispatial neglect; PPV, positive predictive value; NPV, negative predictive value; FOP, field of perception; FOR, field of regard; SR, success rate; RT, reaction time
